# Phase Transformations in a Human Tooth Tissue at the Initial Stage of Caries

**DOI:** 10.1371/journal.pone.0124008

**Published:** 2015-04-22

**Authors:** Pavel Seredin, Dmitry Goloshchapov, Tatiana Prutskij, Yury Ippolitov

**Affiliations:** 1 Department of Solid State Physics and Nanostructures, Voronezh State University, Voronezh, Russia; 2 Instituto de Ciencias, Benemérita Universidad Autónoma de Puebla, Puebla, Mexico; 3 Department of Preventive Dentistry, Voronezh State Medical Academy, Voronezh, Russia; University of Oulu, FINLAND

## Abstract

The aim of the paper is to study phase transformations in solid tissues of the human teeth during the development of fissure caries by Raman and fluorescence microspectroscopy. The study of the areas with fissure caries confirmed the assumption of the formation of a weak interaction between phosphate apatite enamel and organic acids (products of microorganisms). The experimental results obtained with by Raman microspectroscopy showed the formation of dicalcium phosphate dihydrate - CaHPO_4_-2H_2_O in the area of mural demineralization of carious fissure. A comparative analysis of structural and spectroscopic data for the intact and carious enamel shows that emergence of a more soluble phase - carbonate-substituted hydroxyapatite - is typical for the initial stage of caries. It is shown that microareas of dental hard tissues in the carious fissure due to an emerging misorientation of apatite crystals have a higher fluorescence yield than the area of the intact enamel. These areas can be easily detected even prior to a deep demineralization (white spot stage) for the case of irreversibly changed organomineral complex and intensive removal of the mineral component.

## Introduction

Among the objectives of the establishment of modern dentistry, the mechanisms of formation of dental caries is one of the main research trends, in other words, preventing the development of disease in the early stages of its emergence—is a highly topical issue.

It is well known that the first sign of the caries process is a spot, with a size and color (of which) being substantially altered over time due to the transformations in the structure and chemical composition of enamel. Intense ion substitutions are known to occur in the apatite crystals comprising tooth enamel at the level of elementary cells. Proceeding of the ion substitutions leads to changes in the phase composition of enamel apatite mineral complex and, consequently, to the violation of dynamic equilibrium in the mechanism of mineral metabolism. As a result it becomes a prerequisite to the formation of dental caries [1].

While enamel apatite formation occurs in accordance with the following well-known reaction:

$$\text{Ca}{\left({\text{HPO}}_{4}\right)}_{2}\cdot 2H_{2}O\to {\text{Ca}}_{\text{8}}{{\text{(PO}}_{\text{4}})}_{6}\cdot 5H_{2}O\to {\text{Ca}}_{\text{10}}{{\text{(PO}}_{\text{4}})}_{6}{\left(OH\right)}_{2}$$ (1)

i.e. dicalcium phosphate dihydrate (DCPD) -> ortho calcium phosphate -> hydroxyapatite, chemical reactions that occur in tooth enamel in the process of tooth decay are much more complicated. Substituted hydroxyapatite (HAP), the main mineral component of tooth enamel, can behave quite unpredictably when dissolved. This is not only the ability of HAP to isomorphic substitutions, but also the possibility to participate in the formation of a broad class of calcium phosphates, the solubility of which is higher than that of HAP tooth enamel.

Hence in a number of works based on theoretical ideas it is suggested that at the early stages of dental caries enamel demineralization under the influence of acid deposition should exert the occurrence of dicalcium phosphate dihydrate and fluorapatite (FAP) [2, 3, 4]. The analysis of phosphates solubility diagrams [5, 6] shows that hydroxyapatite is the most stable form of calcium phosphate (except fluoroapatite) at pH close to neutral value. Dicalcium phosphate dihydrate according to the diagram [5] appears to be more stable in an acidic environment outside of a singular point where two solid solutions are in the equilibrium (enamel and dicalcium phosphate dihydrate). Considering these facts, according to theoretical calculations it was shown that the probability of such phase transformations increases at low pH values (4.3–5.5). However, such transformation chain has still not been validated experimentally by a direct research of caries process development. It should be noted that during the development of the caries process elemental composition of the affected area, i.e. the degree of substitution, varies with the depth of penetration into enamel caries [7]. It means that dental caries is not merely a chain of chemical reactions in the processes of dissolution of hydroxyapatite. They are likely to experience re-precipitation according to selective dissolution of minerals. It means possible feedbacks of HAP crystal growth processes in the affected areas of hard dental tissues. Analyzing the above-stated, it can be argued that organic matter plays much more significant role of in the process of dental caries and that requires a thorough comprehensive study, since as organic component it can act as an inhibitor of hydroxyapatite crystal growth.

As it was shown previously [8–11], the most convenient method to study a molecular structure, as well as fine structural properties of the different kinds of objects, including biological ones is Raman microspectroscopy. Using this technique of nondestructive testing, researchers can study the molecular vibrational bands of biological objects, which are specific to particular chemical groups. Along with IR spectroscopy [8], this method allows to identify both structural and quantitative composition of a substance. Now a new technique based on polarized Raman spectroscopy was demonstrated for detecting early dental caries on the extracted human teeth. Sound tooth enamel exhibited a strong Raman polarization anisotropy whereas early caries consistently showed a lower degree of Raman polarization anisotropy. In particular, for sound enamel, the Raman peak arising from the symmetric ν_1_ vibration of PO_4_ at 959 cm^-1^ is strongly polarized. This is in contrast to the spectra of carious lesions that displayed weaker polarization dependence at 959 cm-1. Such difference in the degree of Raman polarization anisotropy allows to make a distinction between early dental caries and sound enamel [9–11].

It should be noted that there are a lot of works dealing with the use of Raman spectroscopy for analyzing the chemical and phase composition of the human teeth. However, no attempt has been made for the experimental study of phase transformations during the development of caries.

At the same time, there is also an insistent issue of detection of the emerging caries. The most developed technology available today is based on fluorescence spectroscopy. Using the fluorescence of dental hard tissues, which appears due to irradiation of enamel with laser light of 488 nm wavelength, it is possible to visualize areas of demineralization, which appear as dark spots on the surface of the tooth. The use of light with the wavelength of 633 nm also allows for a good visualization of a bacterial plaque on the tooth surface [12].

Therefore, the aim of our work was to study phase transformations in the hard tissues of the human teeth during the development of fissure caries by Raman and fluorescence microspectroscopy.

## Materials and Methods

**Fig 1:**
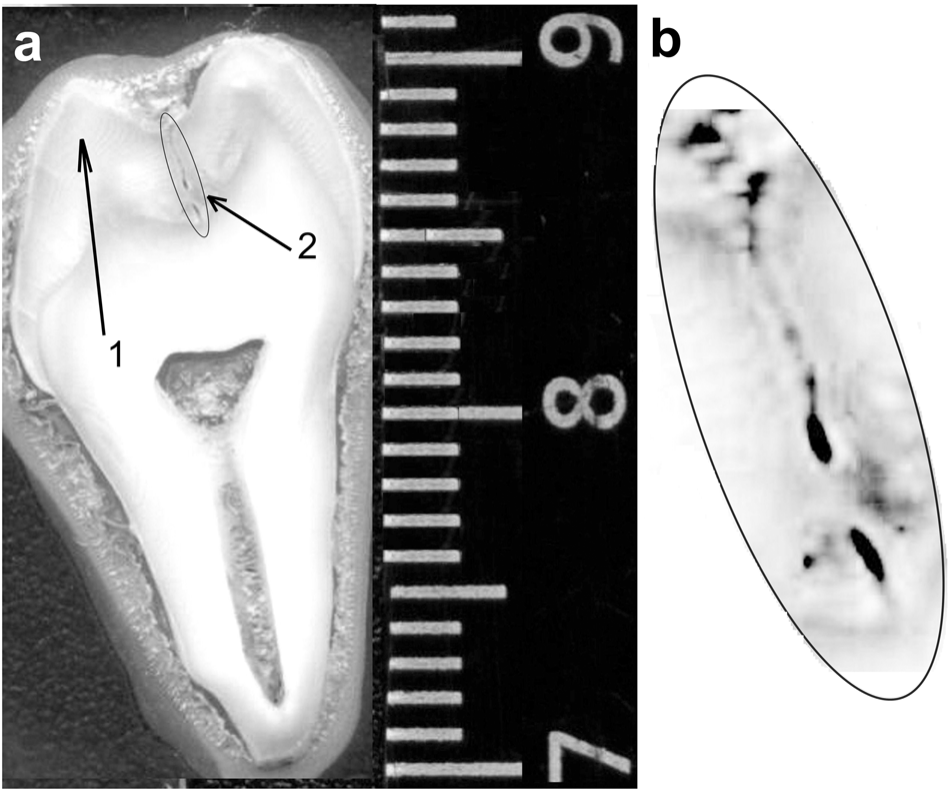
Frontal slice of tooth: total view of the tooth (A); fissure carious canal (B). Frontal slice of the tooth where the investigated areas are indicated (right scale in centimeters); 1–2 are the points that were used for the study.

We investigated ten caries teeth samples (with carious fissures). Permanent masticatory human teeth were removed of European descent patients in the age of 18–25 years. A criterion for selection of the samples was the presence of infected fissures on the masticatory surface of molars and premolars. Voronezh State University Ethics Committee approved this study, approval number 001.000–2012. Teeth were prepared in the following way. Initially, the teeth were washed in flowing water, purified from plaque, and then surfaces were dried with an absorbent. Next, using a microtome, the teeth were cleaved and we got slices with the thickness of ~ 1 mm. The resulting microsections were glued to a glass plate of 2 mm thick using an acrylate adhesive. Fig 1A presents a photograph of the typical sample to be analyzed, Fig 1B shows the area of carious fissures in more detail. It should be noted that this Fig 1B is presented in grayscale, therefore the deeper the area in carious fissure the darker its image in the Fig 1B.

Raman spectra in backscattering geometry were measured at room temperature using a conventional experimental setup, which includes a close-cycle He cryostat, a TRIAX550 monochromator, and a liquid- nitrogen-cooled charge-coupled-device (CCD) detector (Princeton Instruments). The laser beam was focused on the sample using a x50 microscope objective, which allowed us to locate the laser spot on the analyzed area within 4–9 μm^2^ limits. Scattered light from the sample was collected and directed to a monochromator and then detected by the CCD matrix (CCD-detector). To measure the Raman spectra, laser excitation power of 40 mW on the sample surface, and acquisition times of 10–20 seconds were used. To reduce the noise, spectrum averaging was set to 5–10. The exciting laser radiation was not polarized, scattered light was also detected without polarization.

The penetration depth of the laser radiation, and, consequently, the effective depth of analysis for Raman scattering can be determined from the ratio λ/2πk where k is an extinction coefficient. For argon laser with λ = 514.5 nm in the analysis of human tooth hard tissues this depth is about 40–60 nm, which makes it reasonable to state that the performed analysis affected only a thin layers of dental tissue.

Particularly, Raman spectra often show an increasing background. Background correction was performed with the method of "rubberband correction" using Bruker optics software. A “rubberband” method determines support points by finding the convex hull for each spectrum. The baselines are then piecewise linear or (smoothing) splines through the support points. Normalization of the Raman spectra was done by the same Bruker optics software.

In our work, the fluorescence spectra were obtained at room temperature on the surface of the samples by means of a standard technique in the same installation as that one of the Raman scattering spectra. In order to focus on the surface, x10 lens was used. Local changes in the fluorescence intensity of the sample surface were successfully analyzed by observing the fluorescence being excited by a 514.5 nm notch filter, which allowed us to visualize well the lighter areas of dental hard tissues with a correspondingly higher fluorescence intensity.

## Results

**Fig 2:**
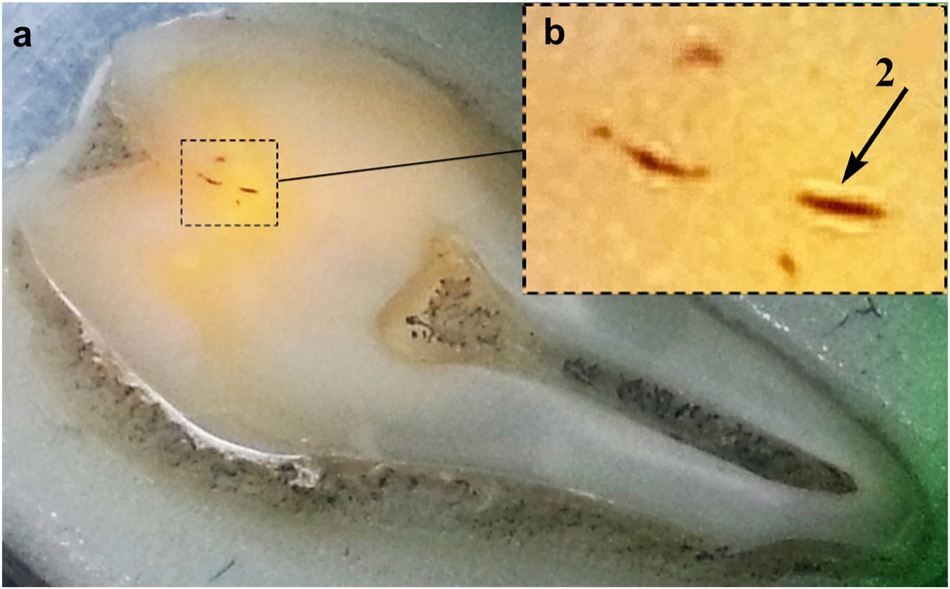
Appearance of the human tooth sample: representation of the human tooth sample irradiated by green laser with a wavelength of 514.5 nm (A); well visualized lighter areas of dental hard tissues (B).

Using the technique of fluorescence excitation of dental hard tissues, we investigated the areas of teeth with enamel carious fissure. Fig 2A shows an image of the typical human tooth sample irradiated by green laser with a wavelength of 514.5 nm. Observation of the excited fluorescence, as previously described, is performed with the use of 514.5 nm notch filter. The analysis of the experimental results shows that the areas around carious fissure (dark spots) are well visualized lighter areas of dental hard tissues (marked by the arrow in the Fig 2B, with a correspondingly higher fluorescence intensity. Intensity increases in carious fissures, different in color and intensity of the radiation of the entire illuminated area intact enamel, has lateral dimensions comparable to the size of the fissure.

It is known that the tooth interprismatic spaces are filled with biopolymers represented, besides neutral glycoproteins [13], by cationic proteins with low molecular weight and a characteristic spectrum of amino acids (lysine, arginine, histidine), as well as by hyaluronic acid. At the initial stages of the caries process in the areas, adjacent to the site of demineralization, biopolymers and hyaluronic acid should be lost that will cause reorganization of the inorganic matrix in the hard tissues of the teeth. This assumption is in a good agreement with the data that we have obtained by means of IR and X-ray microdiffraction spectroscopy [8].

Initial stage of caries is characterized by increase in the number of deformation and valence vibrations for N-C-O, N-H and C = O bounds, decrease in crystallinity index and by the absence of the preferable orientation of hydroxyapatite (HAP) crystals within the affected enamel [8], as opposed to the intact enamel [14].

Therefore enamel microareas adjacent to carious fissure (area of deciduous demineralization) have a higher fluorescence yield than the areas of intact enamel and can be readily detected beforehand. The stage of white spot carious lesions appears when there are irreversible changes taking place in the organo-mineral complex and there is an intense removal of the mineral component.

Based on these results, we found it logical to conduct a detailed study of the molecular structure of micro-organic-enamel complex, which corresponds to the initial phase of caries. Due to the microscopic size of fissures and carious areas and the above features of the objects, the most convenient tool for research of the components of this type is micro-Raman spectroscopy. Micro-Raman was used to characterize dental lesions chemically in this investigation. The study of chemical composition of tooth tissue structures is important in biomedical materials because of the sensitivity of biological systems to the local atomic order [15].

As it was noted previously in Materials and Methods section we have studied 10 typical samples. The obtained spectra from the parts of the intact and carious enamel for all of the samples were of the same type. (they involved similar set of Raman modes insignificantly different from each other). Therefore all of the spectra were averaged in a single-type spectrum.

**Fig 3:**
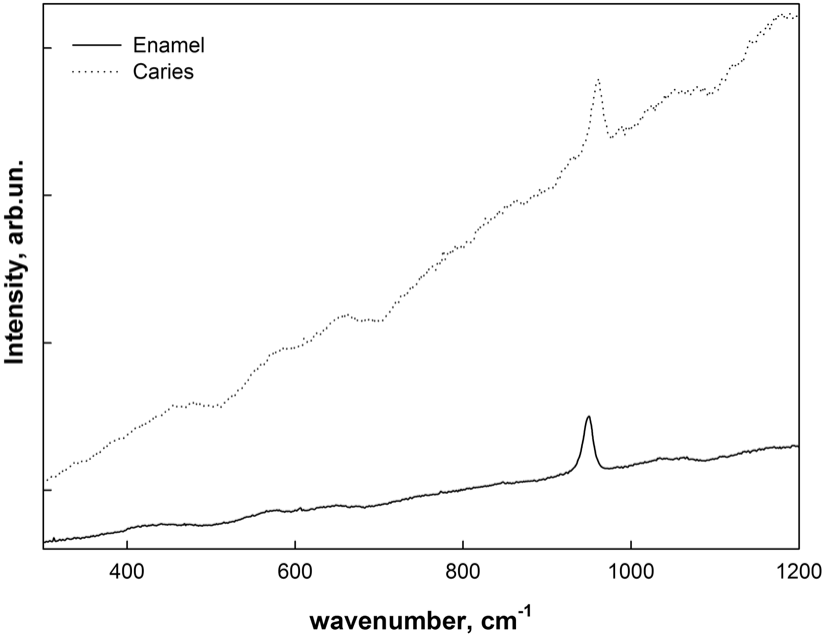
Raman scattering spectra collected from the intact and caries enamel.

Fig 3 shows Raman scattering spectra obtained from the intact enamel as well as a typical spectrum of a microarea of carious fissure with deciduous demineralization (i.e. of the area of intense fluorescence in Fig 2 which is marked by arrow). A note has to be made that the latter spectrum is an average sum of the spectra obtained from different points of intense fluorescence. This helps to exclude the effect of the roughness of the surface on the experimental results and identify the actual differences caused by the phase transformations.

**Fig 4:**
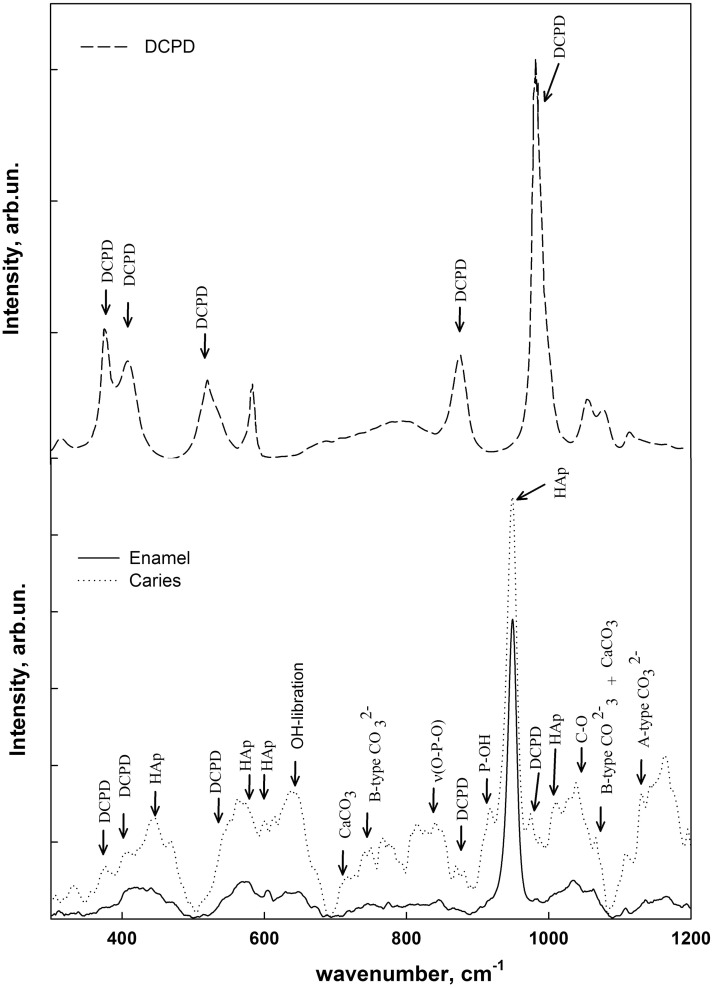
The Raman spectra of tooth enamel after the procedure named as rubberband correction. DCPD Raman Spectra—on top; sound and carious enamel—at the bottom.

As can be seen from the experimental data, the obtained spectra include a very intense fluorescence background from the samples produced by the laser diode using an excitation Raman scattering. Background correction was performed with the use of "rubberband correction" method [16]. Fig 4 shows the typical Raman spectra of intact and carious tooth enamel following the "rubberband correction" and normalization.

The analysis of transformed spectra was conducted using a series of references used in the methods where Raman and FTIR spectroscopy were involved in the examination of an intact human tooth hard tissue, as well as the relevance phosphates participating in the formation of the enamel and dentin [17–21]. A list of active modes and their assignments is shown in (Table 1).

**Table 1:** Active vibration modes in the spectrum of intact enamel and enamel area with the emerging caries process and their assignment to specific molecular groups.

Point 1. Enamel.	Point 2. Caries.	Assignment	Ref.
	380	DCPD	[17]
	410	DCPD	[17]
437	448	$\nu_{2}{\text{PO}}_{\text{4}}^{\text{3-}}$ (HAp)	[9–11], [21]
581	572	$\nu_{4}{\text{PO}}_{\text{4}}^{\text{3-}}$ (HAp)	[9–11], [21]
	607	$\nu_{4}{\text{PO}}_{\text{4}}^{\text{3-}}$ (HAp)	[9–11], [21]
649	647	OH libration (HAp)	[9–11], [21]
	710	CaCO_3_ hydrated	[9–11], [21]
754	766	B-type $\nu_{4}{\text{CO}}_{\text{3}}^{\text{2-}}$ (HAp)	[9–11], [21]
835	841	*ν*(O—P—O)	[9–11], [21]
	877	DCPD	[17]
	927	P-OH stretch (HAp)	[9–11], [21]
960	960	$\nu_{1}{\text{PO}}_{\text{4}}^{\text{3-}}$(HAp)	[9–11], [21]
	986	DCPD (max.int.)	[17]
	1015	$\nu_{3}{\text{PO}}_{\text{4}}^{\text{3-}}$(HAp)	[9–11], [21]
1043	1049	1. $\nu_{3}{\text{PO}}_{\text{4}}^{\text{3-}}$(HAp) 2. DCPD 3. *ν*(C—O)	[17] [9–11], [21]
1072	1072	1. B-type ${\text{CO}}_{\text{3}}^{\text{2-}}$ (HAp)—PO_4_ substitution 2. DCPD 3. CaCO_3_ hydrated	[17] [9–11], [21]
	1117	A-type ${\text{CO}}_{\text{3}}^{\text{2-}}$ (HAp)—OH substitution	[17]

The obtained results have shown that the spectrum includes all of the intact enamel significant fluctuations correlated with the inorganic component of a hard dental tissue, and their frequencies are in excellent agreement with the data of a series of works. The highest intensities in the Raman scattering spectra of intact enamel modes are in the following functional groups: ${\text{PO}}_{\text{4}}^{\text{3-}}$($\nu_{1}{\text{PO}}_{\text{4}}^{\text{3-}}$,$\nu_{2}{\text{PO}}_{\text{4}}^{\text{3-}}$ ,$\nu_{4}{\text{PO}}_{\text{4}}^{\text{3-}}$), OH (OH-libration) and ${\text{CO}}_{\text{3}}^{\text{2-}}$ (correlated with fluctuations B-type substituted hydroxyapatite (i.e., when the group CO_3_ substitutes the group PO_4_).

It should be noted that in the Raman scattering spectrum obtained from the area of deciduous demineralization of enamel apatite in the carious fissure tooth tissue, there is not only the intensity variation of the fundamental vibrations, typical of the spectrum of a healthy enamel, but there is also the occurrence of additional active modes. A simultaneous increase in the intensity of the modes localized near 775 cm^-1^ and 1075 cm^-1^ pertaining to ${\text{CO}}_{\text{3}}^{\text{2-}}$ bonds can be observed, i.e. substitution of the phosphate HAP group in the structure by carbonate ions. There are also oscillations at 1115 cm^-1^ corresponding to ${\text{CO}}_{\text{3}}^{\text{2-}}$ bonds of A-type, substitution of OH-group by carbonate ions in the spectra of carious areas.

However, one of the most interesting features of Raman scattering spectra from the areas of deciduous demineralization in the carious fissure is the presence, along with all the above-mentioned features, of intact enamel a series of low-intensity but distinctly discernible additional vibrational modes is clearly seen, located at ~ 380 cm^-1^, ~ 410 cm^-1^, ~ 875 cm^-1^, ~ 987 cm^-1^ ~ 1050 cm^-1^. The analysis shows that this set of vibrations is characteristic of dicalcium phosphate dihydrate—CaHPO_4_. Fig 4 shows the experimental Raman spectrum of dicalcium phosphate dihydrate, allowing for a clear comparison of the data obtained.

All of the active vibration modes in the spectrum of intact enamel and enamel area with an emerging caries process, as well as their assignment to specific molecular groups, are shown in (Table 1).

## Discussion

The development of caries in hard dental tissues is related to the presence of oral pathogens and their metabolic products—organic acids, among which is the lactic acid. This acid and its residues are directly involved at the beginning (of the process) along with a dissolved organic matrix of enamel, and following that, together with the products of decomposition of organic matter lead to the dissolution of a mineral component [22].

Processes of dissolution and transport of inorganic elements (calcium and phosphorus) of enamel exposed to penetrating of acids into the hard tissue of the tooth are in a way connected with the forces that seek to restore the balance between the concentrations of soluble calcium and phosphorus in a plaque and in the depths of the enamel. The source of these forces is the osmotic pressure in the considered system leading to a rapid leveling while a mutual penetration of solvent molecules through a selectively permeable membrane, which acts as a barrier to the fabric surface of the tooth (dental biofilm) between the oral fluid, rich in trace elements and hard tissue of the tooth. At the initial stage of the caries process solvent molecules (and their acid residues) actively penetrate through biobarrier deep into the enamel under the influence of osmotic forces.

When the acid comes in contact with calcium phosphates, the defining parameters now are the acid strength and its concentration. This are just the factors that determine the degree of conversion of hydroxyapatite crystals; i.e. acidic (CaHPO_4_) or very acidic (Ca_2_(HPO_4_)_2_) calcium phosphates are formed. Since all organic acids are weaker than phosphoric one, decomposition process of the tooth enamel does not occur until quite acidic calcium phosphate is formed instead of hydroxyapatite. Since many organic acids are involved in the process of formation of dental caries, it can be assumed that an acid anion influences the type of the formed compound including calcium. In this case, on the surface of dissolving hydroxyapatite there will be crystallized calcium salts of these acids, or soluble complex compounds are formed [23] and in this case it is possible the formation of acidic calcium phosphates without hydroxyapatite dissolution.

In general, the reaction between the acid and calcium hydroxyapatite, which is the basis of the tooth enamel, is as follows.

$$Ca_{10}{\left(PO_{4}\right)}_{6}{\left(OH\right)}_{2}+8HAn=6CaHPO_{4}+4Ca{\left(An\right)}_{2}+2H_{2}O$$ (2)

where ***An*** represents acid anion. Accordingly, substituting ***An*** for lactic acid *CH*_3_ *CH*(*OH*)*COO* we can obtain the final chemical reaction.

Thus for lactic acid we obtain the following reaction:

$$Ca_{10}{\left(PO_{4}\right)}_{6}{\left(OH\right)}_{2}+8CH_{3}CH(OH)COOH\to 6CaHPO_{4}+4Ca{\left(CH_{3}CH(OH)COO\right)}_{2}+2H_{2}O$$ (3)

As a result, we see the formation of DCPD (brushite), which agrees well with the idea of developing caries, put forward in a series of works [2, 3, 4], as well as with our experimental data.

It should be noted that we observed the formation of ${\text{CO}}_{\text{3}}^{\text{2-}}$bonds of A-type in apatite enamel, which is the result of OH-group substitution by carbonate-ion through incipient caries process. This is consistent both with the ultimate reaction of dissolution of hydroxyapatite to form dicalcium phosphate dihydrate, and with the results that we obtained using the methods of X-ray microdiffraction [14]. According to the data obtained in the area of carious fissure, there is a considerable decrease in crystallite size compared to the area of the intact enamel, which is quite typical of the formation of carbonate-substituted apatite [3, 24–25]

However, apart from the processes of apatite enamel dissolution and the formation of less stable calcium phosphate, the influence of acids on the formation of hydroxyapatite enamel leads to the occurrence of hydrated layer which has been shown in several studies [26, 27]. A partial dissolution of enamel apatite in the reaction with an acid can mean the loss of just OH-groups [28], and since the concentration of acids that affect tooth enamel is not constant over time, while hydroxyapatite is most likely to represent the final phase, the short-term restoration of the structure to form a carbonate-substituted hydroxyapatite of A-type substitution is likely at the beginning of the caries process as well.

## Conclusions

The research of fluorescence properties of dental hard tissues, including areas affected to caries, confirms the assumptions on the changes taking place both in mineral and organic components in the areas of enamel where caries takes place.

The comparative analysis of structural and spectroscopic data for the intact and carious enamel shows that in the area of deciduous demineralization of carious fissure the phase composition of the mineral component changes substantially. The identified change in the redistribution of vibration modes of ${\text{PO}}_{\text{4}}^{\text{3-}}$and ${\text{CO}}_{\text{3}}^{\text{2-}}$ groups in the intact enamel and appearance of A-type substitution of OH-groups by carbonate-ion in the area of carious process agrees well with the assumption that more soluble phase—carbonate-substituted hydroxyapatite occurs.

The mechanisms of weak phosphate formation due to interaction between apatite enamel and some organic acids (products of microorganisms vial acrivity) concerned in the work are confirmed experimentally. The results obtained by means of Raman microspectroscopy indicate at the formation of DCPD—CaHPO_4_ in the area of carious fissure.

In our opinion, studying the role of organic matter in the process of protecting HAP crystals during dissolution of the dental hard tissues and / or facilitating the redeposition of hydroxyapatite crystals in the process of restoration of tooth enamel is a very promising task to be addressed for the future research.

## References

[1] Mann S (2001) Biomineralization: principles and concepts in bioinorganic materials chemistry. New York, Oxford University Press.

[2] Robinson C, Shore RC, Brookes SJ, Strafford S, Wood SR, Kirkham J. (2000) The Chemistry of Enamel Caries. Critical Reviews in Oral Biology & Medicine 11(4): 481–495. 10.2147/OTT.S46933 11132767

[3] Moreno EC, Aoba T. (1990) Solubility of human enamel mineral. J Biology Buccale 18(3): 195–201. 2174870

[4] Aoba T (2004) Solubility properties of human tooth mineral and pathogenesis of dental caries. Oral Dis 10(5): 249–257. 15315640 10.1111/j.1601-0825.2004.01030.x

[5] Margolis HC, Zhang YP, Lee CY, Kent RL Jr, Moreno EC (1999) Kinetics of enamel demineralization in vitro. J. Dent. Res. 78(7): 1326–1335. 10403460 10.1177/00220345990780070701

[6] Veresov AG, Putlaev VI, Tretyakov UD (2004) Chemistry of inorganic biomaterials on the basis of calcium phosphates. The Russian chemical journal of the society named after D.I. Mendeleev 48(4): 52–64.

[7] Robinson C, Kirkham J, Shore R (1995) Dental enamel: formation to destruction. Boca Raton, CRC Press.

[8] Seredin P, Kashkarov V, Lukin A, Ippolitov Y, Julian R, Doyle S (2013) Local study of fissure caries by Fourier transform infrared microscopy and X-ray diffraction using synchrotron radiation. Journal of Synchrotron Radiation 20(5): 705–710. 10.1107/S0909049513019444 23955033

[9] Ko AC, Choo-Smith LP, Hewko M, Lorenzo L, Sowa MG, Dong CC et al (2005). Ex vivo detection and characterization of early dental caries by optical coherence tomography and Raman spectroscopy. J. Biomed. Opt. 10 (3), 031118 16229643 10.1117/1.1915488

[10] Ko AC, Choo-Smith LP, Hewko M, Sowa MG, Dong CC, Cleghorn B (2006) Detection of early dental caries using polarized Raman spectroscopy. Opt. Express. 14 (1): 203–215. 19503331 10.1364/opex.14.000203

[11] Ko AC, Hewko M, Sowa MG, Dong CC, Cleghorn B, Choo-Smith LP (2008) Early dental caries detection using a fibre-optic coupled polarization-resolved Raman spectroscopic system. Opt. Express. 16 (9): 6274–6284. 18545331 10.1364/oe.16.006274 PMC2435308

[12] Gimenez T, Braga MM, Raggio DP, Deery C, Ricketts DN, Mendes FM (2013) Fluorescence-Based Methods for Detecting Caries Lesions: Systematic Review, Meta-Analysis and Sources of Heterogeneity. PLoS ONE 8(4): e60421 10.1371/journal.pone.0060421 23593215 PMC3617206

[13] Ippolitov Y, Ippolitov I, Seredin P (2014) Morphology of the human dental enamel. Indian Journal of Dentistry: 5 (Suppl.): 135–139.

[14] Kashkarov VM, Goloshchapov DL, Rumyantseva AN, Seredin PV, Domashevskaya EP, Spikavova IA et al (2011) X-ray diffraction and IR spectroscopy investigation of synthesized and biogenic nanocrystalline hydroxyapatite. Journal of Surface Investigation. X-ray, Synchrotron and Neutron Techniques 5(6): 1162–1167.

[15] Zavala-Alonso V, Loyola-Rodríguez JP, Terrones H, Patiño-Marín N, Martínez-Castañón GA, Anusavice K (2012) Analysis of the molecular structure of human enamel with fluorosis using micro-Raman spectroscopy. Journal of Oral Science. 54 (1): 93–98. 22466892 10.2334/josnusd.54.93

[16] Lieber CA, Mahadevan-Jansen A (2003) Automated Method for Subtraction of Fluorescence from Biological Raman Spectra. Applied Spectroscopy 57(11): 1363–1367. 14658149 10.1366/000370203322554518

[17] Tsuda H, Arends J (1997) Raman spectroscopy in dental research: a short review of recent studies. Adv. Dent. Res. 11(4): 539–547. 9470515 10.1177/08959374970110042301

[18] Reyes-Gasga J, Martínez-Piñeiro EL, Rodríguez-Álvarez G, Tiznado-Orozco GE, García-García R, Brès FE (2013) XRD and FTIR crystallinity indices in sound human tooth enamel and synthetic hydroxyapatite. Materials Science and Engineering: C 33(8): 4568–4574. 24094161 10.1016/j.msec.2013.07.014

[19] Markovic M, Fowler BO, Tung MS (2004) Preparation and comprehensive characterization of a calcium hydroxyapatite reference material. Journal of Research of the National Institute of Standards and Technology 109(6): 553. 27366634 10.6028/jres.109.042 PMC4856200

[20] Schulze KA, Balooch M, Balooch G, Marshall GW, Marshall SJ (2004) Micro-Raman spectroscopic investigation of dental calcified tissues. J Biomed Mater Res A 69(2): 286–293. 15058001 10.1002/jbm.a.20130

[21] Tsuda H, Arends J (1994) Orientational micro-Raman spectroscopy on hydroxyapatite single crystals and human enamel crystallites. J. Dent. Res. 73(11): 1703–1710. 7983256 10.1177/00220345940730110501

[22] Gross EL, Beall CJ, Kutsch SR, Firestone ND, Leys EJ, Griffen AL (2012) Beyond Streptococcus mutans: Dental Caries Onset Linked to Multiple Species by 16S rRNA Community Analysis. PLoS ONE 7(10): e47722 10.1371/journal.pone.0047722 23091642 PMC3472979

[23] Pan H, Darvell BW (2010) Effect of Carbonate on Hydroxyapatite Solubility. Crystal Growth & Design 10(2): 845–850.

[24] Kapolos J, Koutsoukos PG (1999) Formation of Calcium Phosphates in Aqueous Solutions in the Presence of Carbonate Ions. Langmuir 15(19): 6557–6562.

[25] Xue J, Zhang L, Zou L, Liao Y, Li J, Xiao L et al (2008). High-resolution X-ray microdiffraction analysis of natural teeth J. Synchrotron Rad. (2008). 15, 235–238 10.1107/S0909049508003397 18421147 PMC2394821

[26] Rey C, Combes C, Drouet C, Sfihi H, Barroug A. (2007) Physico-chemical properties of nanocrystalline apatites: Implications for biominerals and biomaterials. Materials Science and Engineering: C 27(2): 198–205.

[27] Farlay D, Panczer G, Rey C, Delmas PD, Boivin G (2010) Mineral maturity and crystallinity index are distinct characteristics of bone mineral. Journal of Bone and Mineral Metabolism 28(4): 433–445. 10.1007/s00774-009-0146-7 20091325 PMC2958843

[28] Kon M, Hirakata LM, Miyamoto Y, Kawano F, Asaoka K (2002) Surface-layer modification of hydroxyapatite ceramic with acid and heat treatments. Dent Mater J 21(2): 170–180. 12238785 10.4012/dmj.21.170

